# A New Three-Way Translocation *t*(4;11;7)(q21;q23;q22) in a Mixed-Phenotype Acute Leukemia

**DOI:** 10.1155/2011/148482

**Published:** 2011-11-10

**Authors:** Hirotaka Takasaki, Takayoshi Tachibana, Masatsugu Tanaka, Atsuo Maruta, Yoshiaki Ishigatsubo, Heiwa Kanamori

**Affiliations:** ^1^Department of Hematology, Kanagawa Cancer Center, 1-1-2 Nakao, Asahi-ku, Yokohama 241-0815, Japan; ^2^Department of Internal Medicine and Clinical Immunology, Yokohama City University Graduate School of Medicine, 3-9 Fukuura, Kanazawa-ku, Yokohama 236-0004, Japan

## Abstract

A 68-year-old man was admitted to our hospital in September 2008 because of a left-sided chest pain. Bone marrow examination showed that 85.5% of leukemic cells were positive for myeloperoxidase (MPO) and were negative for esterase stain. Flow cytometric analysis (FCM) revealed the expression of CD19, CD79a, CD13, CD33, CD34, and HLA-DR on the blasts. Cytogenetic analysis of bone marrow cells using the G-banding technique demonstrated 47, XY, +X, *t*(4;11;7)(q21;q23;q22) in five of the 20 analyzed cells. The patient was diagnosed as having mixed biphenotypic acute leukemia according to the European Group for Immunologic Classification of Leukemia criteria. Mixed-phenotype acute leukemia is a rare, difficult to diagnose entity. Whether patients with mixed-phenotype acute leukemia should be treated with regimens designed for acute myeloid leukemia, acute lymphoblastic leukemia, or both remains unclear.

## 1. Introduction

Acute leukemias of ambiguous lineage are a rare disease accounting for about 3–5% of all acute leukemia [1–3]. It is known that poor-prognosis chromosomal abnormalities such as Philadelphia chromosome and mixed-lineage leukemia (MLL) gene mutations are common in a leukemia subtype expressing B-cell precursor and myeloid cell surface antigens. Therefore, according to the WHO classification of lymphoid neoplasms, the subgroups with *t*(9;22)(q34;q11) and *t*(v;11q23) are classified separately as mixed-phenotype acute leukemia with *t*(9;22)(q34;q11.2), BCR-ABL1, or with *t*(v;11q23), MLL rearranged, respectively. Additional chromosomal abnormalities associated with rearrangements of 11q23 have been reported, but the clinical significance of another chromosome being involved in MLL is still unclear. Here, we report a patient who had mixed-phenotype acute leukemia caused by a new three-way translocation *t*(4;11;7)(q21;q23;q22) involving the *MLL* gene.

## 2. Case Report

**Figure 1:**
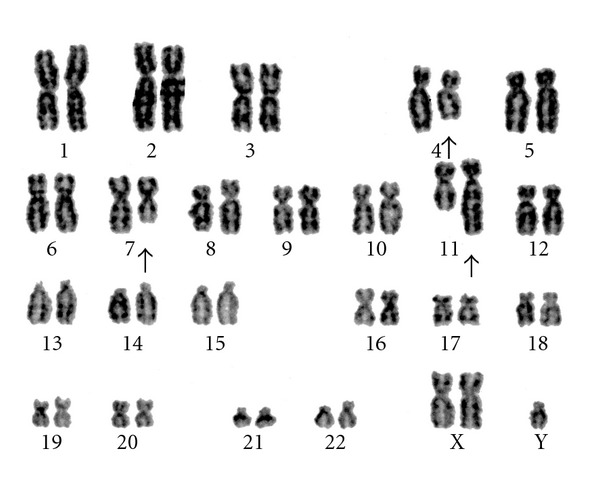
Cytogenetic analysis of bone marrow by the G-banding technique revealed *t*(4;11;7)(q21;q23;q22).

**Figure 2:**
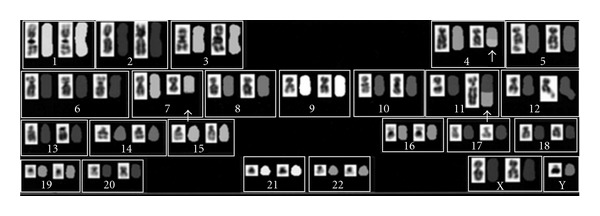
Spectral karyotyping fluorescence in situ hybridization revealed *t*(4;11;7)(q21;q23;q22).

In September 2008, a 68-year-old man visited his local doctor with the chief complaint of left-sided chest pain. Hematology tests examination showed a white blood cell (WBC) count of 133.1 × 10^9^/L with 85.5% blasts. The patient was referred to our hospital for further examination and treatment. Laboratory studies revealed a WBC count of 146.5 × 10^9^/L with 70.0% of blasts, hemoglobin of 12.4 g/dL, and platelet count of 50 × 10^9^/L. The serum lysozyme level was elevated. Bone marrow examination revealed 68.0% of blasts, which were positive for myeloperoxidase (MPO) and negative for esterase staining. Surface antigen analysis by flow cytometry (FCM) indicated that 82.5, 66.8, 23.4, 43.7, 85.9, and 90.7% of the blasts were positive for CD19, CD79a, CD13, CD33, CD34, and HLA-DR, respectively, while the blasts were negative for CD2, CD3, CD5, CD7, CD8, CD10, CD20, and MPO (using a cutoff of 20%). Monocytic markers such as CD11c, CD14, CD36, and CD64 were not tested by FCM. Screening for leukemia-associated fusion genes resulted in identification of the *MLL-AF4* fusion gene. Cytogenetic analysis of bone marrow cells using the G-banding technique demonstrated 47, XY, +X, *t*(4;11;7)(q21;q23;q22) in five of the 20 analyzed cells (Figure 1), and spectral karyotyping confirmed this alteration (Figure 2). The karyotype of the other 13 analyzed cells was 48, sl, +6 and 2 analyzed cells was 46, XY. Application of the European Group for Immunologic Classification of Leukemia (EGIL) criteria produced a diagnosis of biphenotypic acute leukemia, because the score for both B-lymphoid (CD19 and CD79a) and myeloid lineages (CD13 and CD33) was higher than two points. He received induction chemotherapy (daunomycin 40 mg/m^2^ on days 1–5 and cytarabine 80 mg/m^2^ on days 1–7) but failed to achieve remission. Subsequently, the patient was treated with other chemotherapy regimens, including high-dose cytarabine and combination chemotherapy for acute lymphocytic leukemia (ALL). However, complete remission was not achieved, and he died of cerebral infarction in June 2009.

## 3. Discussion

*MLL* gene rearrangements are frequently observed in pediatric leukemias and topoisomerase II inhibitor therapy-related leukemias. The *MLL* gene is located on chromosome 11q23, a typical breakpoint in chromosomal translocations, and it can undergo reciprocal translocation with more than 50 different partner genes, leading to the formation of chimeric genes [4, 5]. In particular, *t*(4;11)(q21;q23) is a recurrent abnormality in ALL although it may also be present in acute myelogenous leukemia (AML) and in mixed phenotype acute leukemia. This translocation is believed to result in malfunction of two genes downstream of the *MLL* gene, which are the *HOXA9 *and *MEIS 1* genes involved in hematopoiesis and hematopoietic stem cell function, leading to the development of leukemia [6].

The clinical significance of additional chromosomal abnormalities in *MLL *gene-associated leukemia is controversial. Moorman et al. investigated the effects of *MLL* gene abnormalities alone versus *MLL* gene abnormalities combined with other chromosomal abnormalities on the behavior and prognosis of ALL and reported that the most frequent additional chromosomal abnormalities were those involving chromosomes 6, 12, and X, which had no influence on the prognosis [7]. On the other hand, Tauchi et al. stated that the presence of additional chromosomal abnormalities, including three-way translocation, was associated with disease progression and a poor prognosis in infants with ALL [8]. The present patient had a translocation at 7q22 in addition to *t*(4;11)(q21;q23). The same chromosome abnormalities detected in the present case, including three-way translocation of MLL-AF4, have been reported by Raffini et al. [9]. Three-way or more frequent chromosomal translocations have been reported in 1.8% of patients with the 11q23 translocation [10]. The *MLL5* gene has been identified as a candidate leukemia suppressor gene located in 7q22, a chromosomal region that is frequently deleted in patients with aggressive AML [11, 12]. Moreover, recent experimental studies have indicated that the *MLL5* gene is associated with regulation of normal hematopoiesis [13, 14]. Although FISH analysis for *MLL5* gene was not tested in our patient, it is speculated that the three-way translocation including 7q22 was related to leukemogenesis and/or proliferation of leukemic cells in line with the literatures.

 In conclusion, we reported a rare case of mixed-phenotype acute leukemia associated with three-way translocation: *t*(4;11;7)(q21;q23;q22). The influence of additional chromosome abnormalities in *t*(4;11) is unclear in this case. Investigation of similar cases will be necessary to clarify the clinical significance of three-way translocations involving the *MLL *gene.

##  Conflict of Interests

The authors state that they have no conflict of interests.
